# Microenvironment-responsive nanomedicines: a promising direction for tissue regeneration

**DOI:** 10.1186/s40779-024-00573-0

**Published:** 2024-10-21

**Authors:** Yuan Xiong, Bo-Bin Mi, Mohammad-Ali Shahbazi, Tian Xia, Jun Xiao

**Affiliations:** 1 Department of Orthopedics, Tongji Hospital, Tongji Medical College, Huazhong University of Science and Technology, Wuhan, 430030 China; 2 Department of Orthopedics, Union Hospital, Tongji Medical College, Huazhong University of Science and Technology, Wuhan, 430022 China; 3 Hubei Province Key Laboratory of Oral and Maxillofacial Development and Regeneration, Wuhan, 430022 China; 4 Department of Biomaterials and Biomedical Technology, Personalized Medicine Research Institute (PRECISION), University Medical Center Groningen (UMCG), University of Groningen, Ant. Deusinglaan 1, Groningen, 9713 AV The Netherlands

**Keywords:** Nanomedicine, Microenvironment, Immunomodulation, Tissue regeneration, Bone, Repair

## Abstract

Severe tissue defects present formidable challenges to human health, persisting as major contributors to mortality rates. The complex pathological microenvironment, particularly the disrupted immune landscape within these defects, poses substantial hurdles to existing tissue regeneration strategies. However, the emergence of nanobiotechnology has opened a new direction in immunomodulatory nanomedicine, providing encouraging prospects for tissue regeneration and restoration. This review aims to gather recent advances in immunomodulatory nanomedicine to foster tissue regeneration. We begin by elucidating the distinctive features of the local immune microenvironment within defective tissues and its crucial role in tissue regeneration. Subsequently, we explore the design and functional properties of immunomodulatory nanosystems. Finally, we address the challenges and prospects of clinical translation in nanomedicine development, aiming to propose a potent approach to enhance tissue regeneration through synergistic immune modulation and nanomedicine integration.

## Background

Tissue defects, stemming from trauma, disease, or congenital anomalies, can manifest in diverse anatomical structures, such as skin, bones, cartilage, nerves, and organs. These defects lead to a range of complications that significantly influence patient outcomes and pose considerable challenges to modern healthcare [1, 2]. Importantly, tissue defects commonly result in impairments that affect mobility, sensation, and regular functioning of the affected body part [3]. Furthermore, the psychological impact of visible deformities and disabilities arising from tissue loss is profound, often resulting in self-consciousness, social withdrawal, and mental health disorders. Additionally, untreated or inadequately managed tissue defects cause significant risks of complications, including infections, persistent inflammation, and secondary deterioration of tissues [4].

The immune microenvironment, characterized by the intricate interplay among immune cells, cytokines, growth factors, and components of the extracellular matrix (ECM), is central to tissue regeneration [5]. Failure of the immune system to initiate a suitable response disrupts the complex cascade of signaling pathways crucial for tissue regeneration, resulting in prolonged healing periods or even regeneration failure. This dysregulation not only hampers the recruitment of vital immune cells and growth factors but also fosters the buildup of fibrotic tissue, thereby impeding effective tissue remodeling and regeneration [6]. Dysfunctions within this microenvironment can significantly hinder the healing process, leading to chronic inflammation that further complicates matters by disrupting the delicate balance necessary for efficient tissue repair. While persistent inflammation is an inherent aspect of the healing process beyond its acute phase, it can adversely affect tissue regeneration by reshaping the immune microenvironment towards a pro-inflammatory state [7]. To overcome this challenge, a comprehensive approach is urgently required that includes a deep understanding of underlying biological mechanisms, the development of innovative therapeutic strategies, and the optimization of existing treatments to meet the needs of individual patients.

**Fig. 1:**
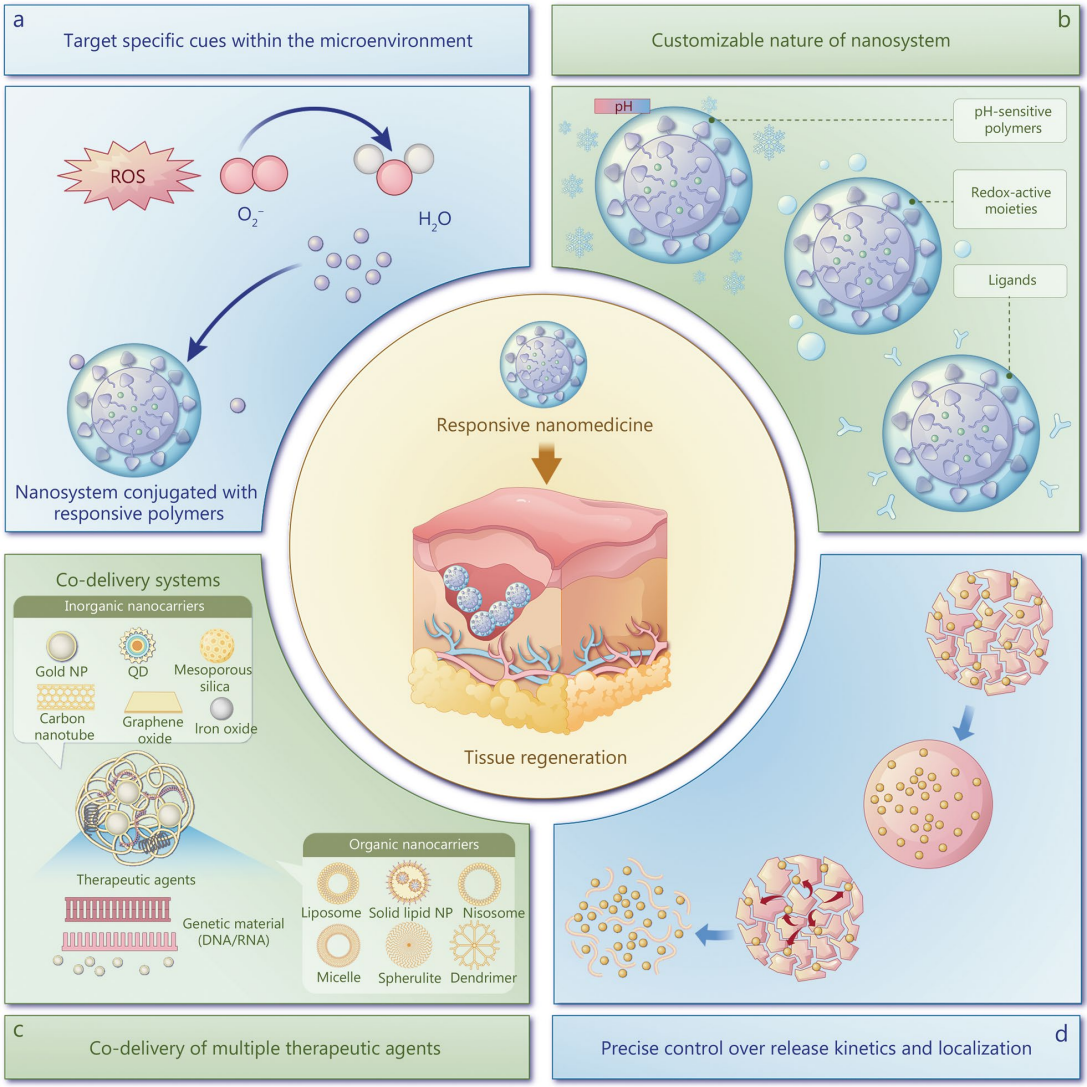
Schematic illustration of microenvironment-responsive nanomedicines for tissue regeneration. ROS reactive oxygen species, QD quantum dots, NP nanoparticles

Microenvironment-responsive nanomedicine is a cutting-edge approach to tissue regeneration and immunomodulation with several distinct advantages. First, the ability to target specific cues within the microenvironment, such as pH, temperature, or enzyme activity, facilitates the precise delivery of therapeutic agents to desired sites, thereby minimizing off-target effects while enhancing efficacy [8, 9]. Through responding to these cues, nanosystems can release therapeutic agents in a controlled manner, optimizing the concentration and duration at the target site, ultimately improving therapeutic outcomes, while reducing systemic side effects. Furthermore, the customizable nature of nanosystems allows for modification in size, shape, surface chemistry, and payload to promote regeneration and address the immunomodulation requirements of specific tissues, offering versatility in clinical applications [10]. Moreover, nanosystems enable the co-delivery of multiple therapeutic agents, such as growth factors, drugs, nucleic acids, and cells, in a single formulation, facilitating synergistic effects and the modulation of multiple pathways involved in tissue repair and immune response regulation [11]. Additionally, precise control over release kinetics and localization helps minimize immune recognition and inflammatory responses, thus promoting tissue healing, while reducing adverse reactions [12, 13] (Fig. 1).

Therefore, this review aims to summarize recent progress in microenvironment-responsive nanomedicine to support tissue regeneration and immunomodulation. We begin by clarifying the unique characteristics of the local immune microenvironment within damaged tissues and its significant functions in tissue regeneration. Next, we investigate the structures and properties of immunomodulatory nanosystems. Finally, we discuss the obstacles and clinical potentials of nanomedicine as an effective strategy for improving tissue regeneration through synergistic integration with immune modulation.

## Immunomodulatory factors in tissue regeneration

Immunomodulatory factors, encompassing cytokines, growth factors, and immune cells such as macrophages and regulatory T cells, play crucial roles in regulating immune responses to injury, thereby promoting tissue repair and regeneration. These factors help orchestrate the complex interplay between inflammation and tissue remodeling and regeneration [14]. Through modulation of the immune response, they can enhance tissue healing, reduce scarring, and promote functional recovery.

### Abnormal immune microenvironment at defect sites

An aberrant immune microenvironment at sites of injury can significantly influence tissue repair, regeneration, and the overall healing processes. Such microenvironments often arise due to various factors, including injury, disease, or dysfunction of the immune system itself. Understanding the intricacies of these aberrant immune responses is crucial for developing targeted therapeutic interventions to improve tissue repair and regeneration.

**Fig. 2:**
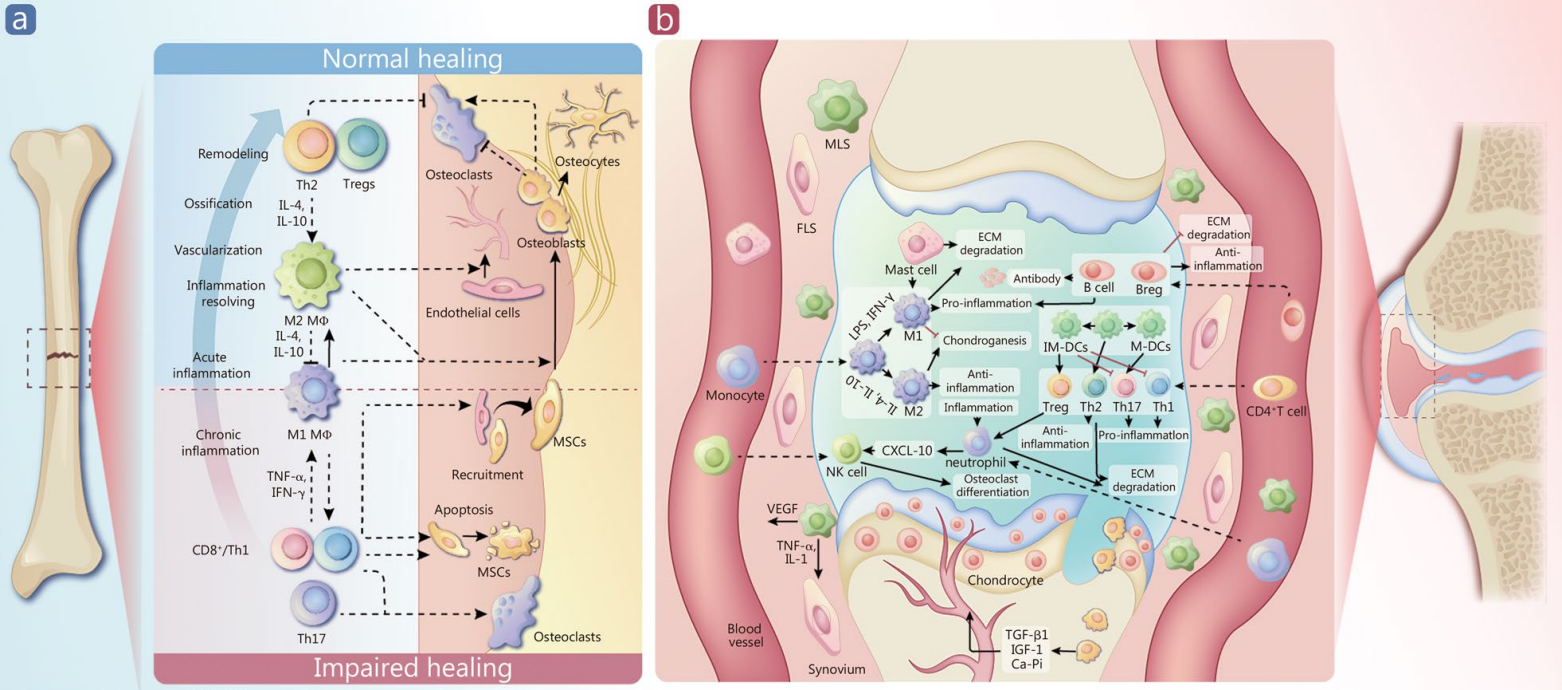
Roles of the immune microenvironment in bone and cartilage regeneration. **a** Immune factors play different roles in successful healing and impaired healing processes. **b** Crucial immune cells and signaling pathways in cartilage regeneration. Th helper T cells, IL interleukin, TNF-α tumor necrosis factor-α, MSCs mesenchymal stem cells, Mø macrophage, MLS multipotent stem cells, FLS fibroblast-like synoviocytes, VEGF vascular endothelial growth factor, CXCL-10 C-X-C motif chemokine ligand-10, LPS lipopolysaccharide, IFN-γ interferon-γ, TGF-β1 transforming growth factor-β1, IGF-1 insulin-like growth factor-1, ECM extracellular matrix, IM-DCs immature dendritic cells, M-DCs mature dendritic cells

The immune response plays a vital role in orchestrating tissue repair and regeneration following injury or insult. In a healthy condition, immune cells such as macrophages, neutrophils, and lymphocytes work synergistically to clear debris, control inflammation, and promote tissue remodeling [15, 16]. However, in abnormal immune microenvironments, this coordinated response is disrupted, leading to dysregulated inflammation, impaired clearance of cellular debris, and altered interactions between immune cells and resident tissue cells. For instance, in chronic inflammatory conditions like rheumatoid arthritis, inflammatory bowel disease, infected bone defect, or cartilage damage, the immune system mistakenly attacks healthy tissues, perpetuating inflammation and hindering proper tissue repair processes [17, 18] (Fig. 2a).

One hallmark of abnormal immune microenvironments at defect sites is the persistence of pro-inflammatory signals and the failure to appropriately resolve inflammation. Chronic inflammation can delay healing by inhibiting the transition from the inflammatory phase to the proliferative and remodeling phases of tissue repair [19]. Inflammatory cytokines, including tumor necrosis factor-α (TNF-α) and interleukin-6 (IL-6), are frequently elevated in these environments, further inducing immune cell activation and exacerbating tissue damage [20]. Additionally, dysregulated activation of immune cells such as pro-inflammatory M1 macrophages can exacerbate tissue injury and impair regeneration (Fig. 2a). The imbalance between pro-inflammatory and anti-inflammatory signals disrupts the delicate equilibrium essential for effective tissue repair, resulting in prolonged healing times and even tissue degeneration [11, 21].

Furthermore, the presence of aberrant immune cells or dysfunctional immune responses can directly impede tissue regeneration processes. At sites of injury, immune cells may adopt alternative phenotypes or undergo abnormal activation, leading to impaired communication with other cellular components involved in tissue repair. For example, regulatory T cells, which typically suppress excessive inflammation and promote tissue tolerance, may exhibit reduced function or abundance under certain pathological conditions, thereby exacerbating immune-mediated damage [14, 21]. Similarly, aberrant activation of fibroblasts or myofibroblasts, driven by persistent inflammatory signals, can lead to excessive collagen deposition and fibrosis, further impeding functional tissue regeneration [22]. The interplay among immune cells, stromal cells, and ECM components is crucial for mediating tissue repair processes; however, disruptions to this balance can have detrimental effects on regeneration outcomes.

In addition to cellular components, the ECM at defect sites undergoes substantial remodeling, which can influence immune cell behavior and tissue repair processes. Abnormalities in ECM composition, such as excessive deposition of fibronectins, collagens, or proteoglycans, can induce a hostile microenvironment that hinders immune cell infiltration and function [23, 24]. Moreover, alterations to ECM stiffness or architecture can directly impact immune cell migration, activation, and polarization [25]. For instance, rigid ECM substrates have been shown to promote the pro-inflammatory phenotypes of macrophages, thereby impairing their ability to resolve inflammation [26]. Conversely, degradation products of the ECM or soluble factors released during tissue injury can modulate immune cell behavior and contribute to the chronicity of inflammation [27]. Thus, the reciprocal interactions between immune cells and the ECM are pivotal in shaping the immune microenvironment at defect sites and ultimately influencing outcomes in tissue repair (Fig. 2b).

### Role of inflammation in tissue defects

Inflammation plays a multifaceted role in tissue defects, constructing a complex series of events that ultimately determine the outcome of tissue repair and regeneration [19]. Initially, inflammation serves as a protective response to injury, aimed at eliminating damaged cells, pathogens, and debris from the site of injury and initiating the repair process [5]. Upon tissue injury, resident immune cells such as macrophages and mast cells release inflammatory mediators, including cytokines, chemokines, and prostaglandins, which promote vasodilation and increase vascular permeability [28]. This results in the recruitment of circulating immune cells like neutrophils and monocytes that contribute to the inflammatory response by phagocytosing debris and secreting additional inflammatory signals at the injury site.

Inflammatory cytokines play crucial roles in coordinating the inflammatory response and modulating the behavior of various cell types involved in tissue repair [29, 30]. TNF-α and IL-1, for example, promote the expression of adhesion molecules on endothelial cells, thereby facilitating the recruitment of leukocytes to the site of injury [31, 32]. Moreover, these cytokines activate local fibroblasts and endothelial cells, leading to the production of ECM components and angiogenic factors essential for tissue repair. Conversely, IL-6 contributes to the activation, polarization, phenotype, and function of immune cells, such as macrophages, during the inflammatory phase of tissue repair [33]. While these pro-inflammatory cytokines are crucial to initiate repair processes, dysregulated or prolonged inflammation can impede tissue regeneration, leading to chronic conditions characterized by fibrosis or degeneration.

In addition to pro-inflammatory signals, the resolution of inflammation is equally essential for effective tissue repair and regeneration. Specialized pro-resolving mediators, including lipoxins, resolvins, and protectins, promote the resolution of inflammation and facilitate the transition to the proliferative and remodeling phases of tissue repair. They exert anti-inflammatory effects by dampening the production of pro-inflammatory cytokines, thereby enhancing the clearance of apoptotic cells and debris, and promoting a shift from the pro-inflammatory M1 to the anti-inflammatory M2 macrophage phenotype [34, 35]. M2 macrophages, in particular, contribute to tissue repair by secreting growth factors, such as transforming growth factor-β (TGF-β) and vascular endothelial growth factor (VEGF), which are instrumental in angiogenesis, collagen deposition, and tissue remodeling [15]. Therefore, timely resolution of inflammation is vital for preventing excessive tissue damage and ensuring optimal tissue repair outcomes.

## Responsive strategies for the tissue microenvironment

Traditional tissue regeneration strategies often fall short in addressing the complexities of the repair process, thereby necessitating innovative approaches. Recent developments in nanobiotechnology have paved the way for the advances of immunomodulatory nanomedicines, which hold promise for enhancing tissue regeneration and restoration. These novel nanomedicines are designed to interact specifically with the local immune microenvironment, which plays a crucial role in the regeneration process. By modulating immune responses at the molecular level, these nanosystems can effectively provide a conducive environment for tissue repair. Key molecular pathways involved in inflammation, such as the nuclear factor-κB (NF-κB) pathway, along with those regulating immune cell differentiation and function, like the Janus kinase/signal transducer and activator of transcription (JAK/STAT) pathway, are targeted to control the immune response. These immunomodulatory nanosystems can deliver anti-inflammatory agents, cytokines, or genetic materials to regulate these pathways, thus reducing inflammation and promoting tissue healing.

**Fig. 3:**
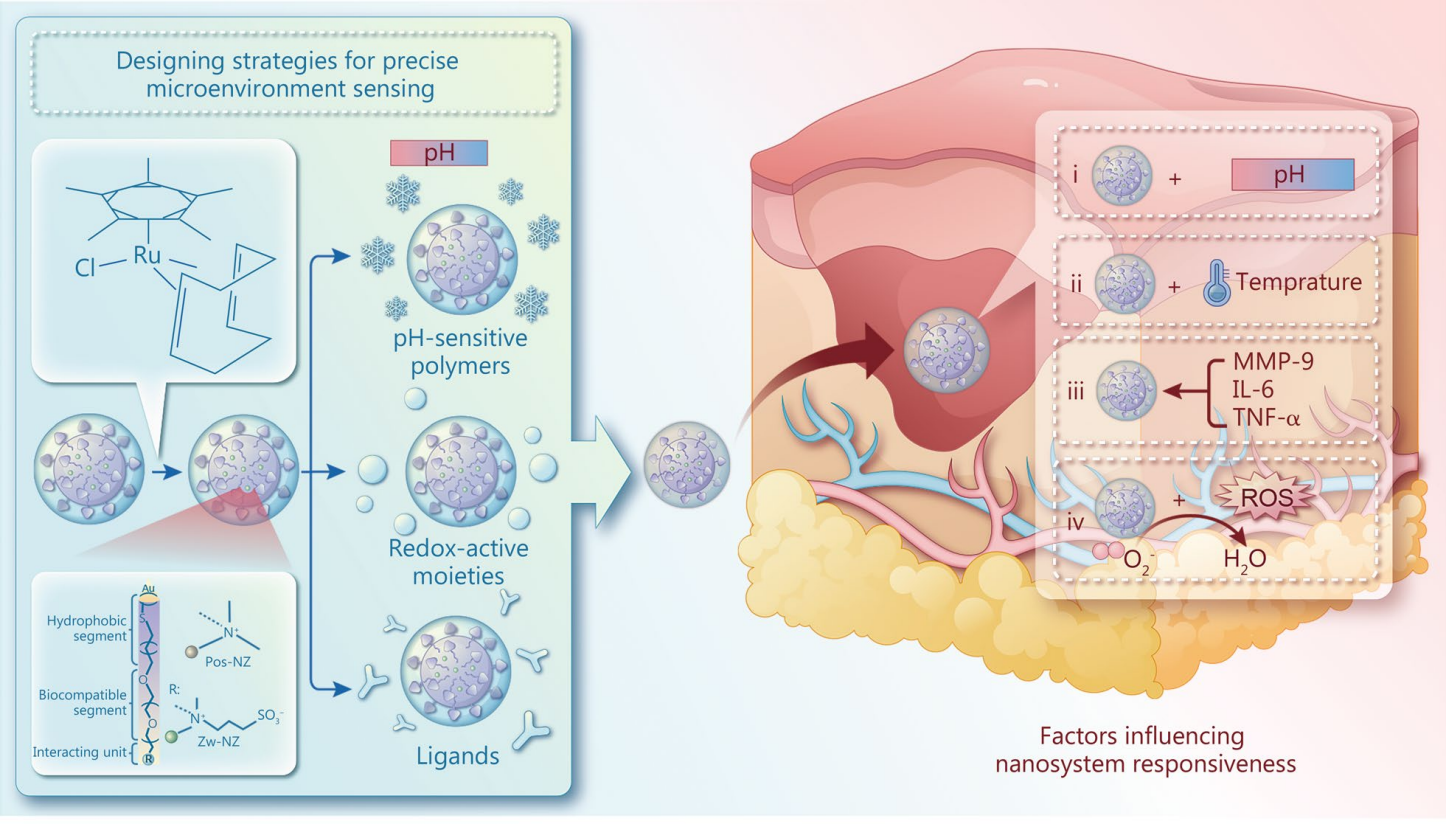
A schematic illustration of responsive strategies for precise microenvironment sensing and tissue regeneration. MMP-9 matrix metalloproteinase-9, IL-6 interleukin-6, TNF-α tumor necrosis factor-α, ROS reactive oxygen species

Additionally, the design of these nanomedicines frequently incorporates stimuli-responsive features, enabling them to release therapeutic agents in response to specific signals within the pathological microenvironment, such as changes in pH, redox conditions, or enzymatic activity. This targeted delivery not only enhances treatment efficacy but also minimizes systemic side effects. Furthermore, the integration of biomimetic materials into these nanosystems can facilitate cellular adhesion, proliferation, and differentiation, essential processes for tissue regeneration. Responsive strategies tailored to the tissue microenvironment represent a cutting-edge approach in regenerative medicine and tissue engineering. Nanotechnology offers precise control over the design, fabrication, and functionalization of nanomaterials, enabling functional interventions at the nanoscale to modulate cellular behaviors, ECM dynamics, and signaling pathways via the sensing of tissue microenvironment (Fig. 3).

### Stimuli-responsive drug delivery

Stimuli-responsive nanosystems provide a dynamic platform for the delivery of immunomodulatory drugs or biologics in response to specific cues within the immune microenvironment. These nanocarriers can be engineered to respond to various stimuli, including alterations in pH, temperature, enzyme activity, or redox status, thereby enabling the triggered release of therapeutic payloads at desired sites [36].

A key advantage of stimuli-responsive nanosystems lies in their ability to exploit various stimuli present at the target site for the controlled release of therapeutic payloads [37, 38]. For instance, pH-responsive liposomes and polymer nanoparticles (NPs) have been engineered to take advantage of the acidic microenvironment of typical inflammatory tissues. Through the incorporation of pH-sensitive components into the design, these nanocarriers remain stable during circulation but undergo rapid drug release upon encountering acidic conditions within damaged tissues [36, 38]. This targeted drug delivery strategy enables selective delivery of therapeutic agents to inflammatory sites while minimizing exposure to healthy tissues, consequently reducing systemic toxicity, and enhancing treatment efficacy.

Similarly, enzyme-responsive nanomaterials present another promising avenue for targeted drug release in response to specific enzymatic activities associated with inflammation or disease [39]. Designing nanocarriers that are sensitive to particular enzymes in the target tissue can achieve precise control over the release of immunomodulatory agents. This approach allows for the modulation of immune responses at sites of inflammation or pathology, offering potential therapeutic benefits for a wide range of conditions, including autoimmune diseases, chronic inflammatory disorders, and tissue injuries [39, 40].

Furthermore, smart biomaterials for tissue engineering that respond dynamically to the microenvironment of damaged or diseased tissues are produced by incorporating stimuli-responsive components into scaffolds or matrices [41, 42]. These biomaterials can release growth factors, cytokines, and other bioactive molecules in a controlled manner to promote tissue regeneration and accelerate the healing process.

### Targeting immune cells

Nanosystems can be engineered to target specific immune cell populations within the microenvironment, facilitating selective modulation of immune responses. In Table 1 [43–50], we summarized various nanomedicine approaches and conducted a comparative analysis of their effectiveness in modulating immune responses. Functionalization of NPs with targeting ligands, such as antibodies or peptides, enables selective binding to the surface receptors of immune cells [51]. For example, targeting specific ligands can enhance targeted delivery of immunomodulatory agents aimed at modulating the functions of T cells, dendritic cells, and macrophages to promote interactions within the immune microenvironment [52].

**Table 1:** Comparison of different nanomedicine approaches in modulating the immune response

Nanomedicine approach	Description	Efficacy	Advantages	References
NPs with surface modifications	Surface-modified NPs (e.g., PEGylation) evade immune recognition	Enhances circulation time; targeted delivery to immune cells	Minimizes off-target effects; enhances therapeutic efficacy	[43, 44]
NPs with immunomodulatory payloads	Loaded with agents (e.g., cytokines, antibodies) to modulate immune responses	Directly influences immune cell behavior; enhances responses	Mediated immune modulation; controlled release minimizes side effects	[45]
NPs as antigen delivery systems	Deliver antigens to APCs for immune activation or tolerance induction	Induces specific immune responses; enhances vaccine efficacy	Mimics natural antigen presentation; potential for targeted immunotherapies	[46–48]
NPs for immune cell engineering	Modify immune cells ex vivo with genetic material (e.g., siRNA, mRNA)	Engineered cells reintroduced to modulate immune responses	Personalized therapies; less immunogenic and more effective targeting of diseases	[49, 50]

*NPs* nanoparticles, *APCs* antigen-presenting cells

Moreover, nanosystems can exploit the phenomenon known as the enhanced permeability and retention (EPR) effect to passively accumulate in inflamed or diseased tissues. The EPR effect is particularly beneficial for targeting inflammatory sites, where abnormal vasculature and leaky blood vessels allow NPs to extravasate and accumulate selectively in affected tissue [53, 54]. Harnessing the EPR effect can further enhance both targeting specificity and accumulation of nanocarriers, thereby maximizing therapeutic efficacy, while minimizing systemic toxicity. The ability to precisely modulate immune responses using nanosystems holds great promise for the development of next-generation immunotherapies. Traditional immunomodulatory therapies are always limited due to systemic toxicity and off-target effects, which may diminish therapeutic utility and cause adverse side effects. In contrast, targeted immunomodulation via nanosystems offers a more precise and controlled therapeutic approach by mitigating off-target effects and reducing the risk of adverse reactions [55].

### Combination therapies

Integration or conjugation of multiple immunomodulatory agents onto nanocarriers can facilitate synergistic effects, overcoming the limitations inherent in single-agent treatments. For instance, the combination of immunomodulatory drugs with anti-inflammatory agents or checkpoint inhibitors can amplify immune responses and improve immunotherapeutic outcomes [56]. This strategic fusion not only enhances efficacy but also minimizes potential adverse effects associated with higher doses of individual agents. Furthermore, the nanoscale dimension of these carriers enables the simultaneous delivery of immunomodulatory agents alongside other therapeutic payloads, such as chemotherapy drugs or nucleic acids [57]. This multifaceted approach holds significant promise for reshaping the landscape of immunomodulation, providing innovative therapeutic alternatives for various diseases.

Moreover, the adaptability of nanosystems extends beyond conventional combination therapy by facilitating precise modulation of drug release kinetics and biodistribution. This level of control allows for the optimization of treatment regimens, ensuring sustained therapeutic concentrations at target sites, while reducing systemic exposure [58]. Additionally, the inherent properties of nanocarriers, such as their ability to bypass biological barriers and target specific cells or tissues, further enhance the therapeutic potential of combination immunomodulatory therapies [59]. Such targeted delivery not only enhances efficacy but also diminishes the risk of off-target effects, thereby fostering safer treatments.

### Biomimetic design

Biomimetic nanosystems that mimic the structure and function of natural immune cells or extracellular vesicles present a novel strategy for modulating immune responses in the microenvironment [10]. These nanocarriers can be engineered to express cell surface markers or ligands that promote interactions with endogenous immune cells, thus facilitating either activation or suppression of immune cells. Moreover, biomimetic nanosystems can leverage endogenous cellular uptake mechanisms, such as phagocytosis and receptor-mediated endocytosis, to deliver immunomodulatory payloads directly to immune cells [60]. For example, exosome-mimetic NPs derived from immune cells can serve as natural carriers for the delivery of therapeutic nucleic acids or proteins aimed to modulate immune responses in cancer therapy [43]. Biomimetic nanosystems provide significant advantages including enhanced biocompatibility, prolonged circulation times, and reduced immunogenicity, making them compelling candidates for immunomodulation across various disease contexts.

## Microenvironment-responsive nanomedicine for tissue regeneration and immunomodulation

Microenvironment-responsive nanomedicine represents a promising advancement in regenerative medicine and immunotherapy, addressing the complex challenges associated with tissue repair and immune modulation. Traditional therapeutic approaches often struggle to achieve precise targeting, sustained release, and effective control of therapeutic interventions within the intricate microenvironments of damaged and diseased tissues. Fortunately, the integration of nanotechnology has propelled the field by providing versatile nano-platforms capable of dynamically sensing, interpreting, and adapting to the dynamic conditions at the tissue level. These innovative nanoscale materials and systems utilize stimuli-responsive behaviors, biomimetic principles, and advanced engineering techniques to facilitate beneficial responses that enhance tissue regeneration while modulating immune reactions. In Table 2 [41, 45, 61–79], we summarized the potential types of nanosystems for microenvironment-responsive applications.

**Table 2:** The potential types of nanosystems for microenvironment-responsive applications

Types of nanosystem	Description	Microenvironmental triggers	Therapeutic application	References
Stimuli-responsive NPs	NPs designed to release therapeutic agents in response to specific physiological triggers	pH, redox conditions, enzymes	Targeted drug delivery and controlled release	[61–63]
Polymeric NPs	Biodegradable and biocompatible NPs made from polymers that can encapsulate drugs, proteins, or nucleic acids	pH, temperature, enzymes	Drug delivery, gene therapy, protein delivery	[64, 65]
Lipid-based NPs	Nanoparticles composed of lipids that can form vesicles, micelles, or solid lipid NPs, ideal for encapsulating hydrophobic and hydrophilic drugs	Enzymes, pH, temperature	Drug delivery, gene therapy, vaccination	[45, 66]
Inorganic NPs	NPs made from metals, metal oxides, or other inorganic materials, often used for their unique optical, magnetic, or electrical properties	pH, redox conditions, enzymes	Imaging, photothermal therapy, drug delivery	[67, 68]
Hybrid NPs	Combination of organic and inorganic materials to leverage the advantages of both, providing multifunctional capabilities	pH, redox conditions, enzymes	Imaging, drug delivery, photothermal therapy	[69, 70]
Dendrimers	Highly branched, tree-like macromolecules with multiple functional end groups, capable of encapsulating or conjugating various therapeutic agents	pH, enzymes, redox	Drug delivery, gene therapy, diagnostic imaging	[71]
Micelles	Amphiphilic molecules that self-assemble into spherical structures in aqueous environments, suitable for encapsulating hydrophobic drugs	pH, enzymes, temperature	Drug delivery, cancer therapy, diagnostic imaging	[72, 73]
Nanogels	Hydrogel nanoparticles that can swell and shrink in response to environmental conditions, allowing the controlled release of encapsulated drugs	pH, temperature, enzymes, redox	Drug delivery, gene therapy, tissue engineering	[74–76]
Carbon-based NPs	Nanoparticles composed of carbon materials like graphene, carbon nanotubes, and fullerenes, known for their high surface area and facile functionalization	pH, redox conditions, temperature	Drug delivery, imaging, photothermal therapy	[77, 78]
Exosomes	Naturally occurring extracellular vesicles that can be engineered to carry therapeutic agents, benefiting from inherent biocompatibility and targeting capabilities	Biochemical signals from target cells	Drug delivery, gene therapy, regenerative medicine	[41, 79]

*NPs* nanoparticles

### Microenvironment-responsive nanomedicine for bone regeneration

Bone regeneration is an exceptionally intricate process, characterized by the dynamic interplay among diverse cellular components, signaling molecules, and ECM. Recently, nanomedicine has emerged as a promising avenue for modulating immune responses and facilitating bone regeneration. This innovative approach involves the development of strategies designed to create a conducive microenvironment that promotes tissue repair by targeting immune cells and manipulating cytokine signaling pathways. Among the key players in this process are macrophages, which exert significant influence over both the inflammatory and reparative phases of bone healing. The delicate balance between pro-inflammatory M1 and anti-inflammatory M2 macrophages is pivotal in orchestrating immune responses that foster tissue repair. Utilizing responsive nanomedicine-based techniques, efforts are focused on regulating macrophage polarization towards the M2 phenotype. This shift is associated with heightened tissue remodeling, angiogenesis, and osteogenesis, all essential elements of successful bone regeneration.

In this context, nanomedicine offers a promising avenue for intervention, to modulate macrophage polarization towards the M2 phenotype to enhance the regenerative process. By employing responsive nanomedicine-based approaches, researchers seek to harness the therapeutic potential of M2 macrophages, which are recognized for their roles in tissue remodeling, angiogenesis, and osteogenesis [80–82]. This targeted modulation of macrophage behavior underscores the innovative strategies being developed to form a supportive microenvironment that fosters and accelerates bone regeneration, thereby offering hope for more effective treatments in the field of regenerative medicine [82, 83].

A prominent strategy for immunomodulation involves engineering nanomaterials capable of regulating the activities of immune cells. For example, glycosylated nano-hydroxyapatites (GHANPs) functionalized with bioactive molecules can target specific receptors on macrophages to promote polarization towards the M2 phenotype [82]. These engineered nanomaterials can also function as carriers for therapeutic agents such as cytokines, growth factors, and small molecules. Cytokines are pivotal in orchestrating immune responses and regulating bone regeneration. Responsive nanomedicine-based approaches aim to manipulate cytokine signaling pathways, thereby fostering a pro-regenerative microenvironment at the injury site. For instance, IL-10, an anti-inflammatory cytokine secreted by M2 macrophages, has been demonstrated to promote osteogenesis while inhibiting bone resorption [83]. The use of engineered nanomaterials for delivery or expression modulation of IL-10 can enhance bone formation and accelerate tissue repair. In addition to targeting immune cells and cytokine signaling pathways, responsive nanomedicine-based approaches also involve the design of biomimetic scaffolds that facilitate tissue growth and regeneration [81, 84]. These scaffolds are typically composed of nanomaterials, such as nano-hydroxyapatite, collagen, or synthetic polymers, which closely mimic the composition and structure of the ECM [84]. Incorporation of bioactive molecules or growth factors into these scaffolds can generate microenvironments that promote cell adhesion, proliferation, and differentiation, ultimately leading to enhanced tissue regeneration.

Responsive nanomedicine-based immunomodulation is promising for the treatment of bone defects and other musculoskeletal disorders. Clinical trials have shown that therapies utilizing nanomaterials can enhance bone healing and regeneration of damaged tissues. However, several challenges remain, including the necessity for standardized protocols, long-term safety assessments, and scalable manufacturing techniques [85, 86]. Future research endeavors should concentrate on optimizing nanomaterial design, elucidating underlying mechanisms of action, and translating findings into clinically applicable therapies for bone regeneration.

### Responsive nanosystems for cartilage regeneration and immunomodulation

Responsive nanosystems exhibit considerable potential in the realm of cartilage regeneration and immunomodulation, providing multifunctional strategies to navigate the intricate pathophysiology associated with cartilage diseases. These innovative systems offer tailored solutions that effectively tackle the complexities inherent in cartilage regeneration, facilitating targeted interventions at the molecular level [87, 88]. Through harnessing responsive nanotechnology, researchers can engineer advanced platforms capable of delivering therapeutic agents precisely to the sites of injury within damaged cartilage tissue, thereby optimizing treatment efficacy while minimizing off-target effects.

Moreover, the multifunctional characteristics of responsive nanosystems allow for the integration of diverse therapeutic modalities, such as drug delivery, gene therapy, and tissue engineering, into a unified platform. This technology convergence fosters comprehensive approaches that not only promote cartilage regeneration but also modulate the immune response, addressing inflammation and immune-mediated processes that contribute to cartilage degradation. Owing to the multifaceted intervention strategy, responsive nanosystems have the potential to advance treatment options in cartilage diseases [89–91].

Osteoarthritis (OA) is characterized by chronic joint inflammation, cartilage degradation, and impaired tissue repair, necessitating the development of innovative therapeutic strategies to alleviate symptoms and promote joint health. Recent reports have highlighted the potential of responsive nanosystems for cartilage regeneration and immunomodulation [92, 93]. One investigation focused on the formulation of macrophage membrane-coated NPs (M2H@RPK) as a novel therapeutic modality for OA [93]. The M2H@RPK nanotherapeutic system aims to simultaneously modulate pro-inflammatory responses and enhance anti-inflammatory pathways within the joint microenvironment by utilizing the immunomodulatory properties of macrophages. Through repolarization of macrophages from the M1 to the M2 phenotype, M2H@RPK effectively reduces the production of pro-inflammatory cytokines, mitigates synovial inflammation, and demonstrates significant therapeutic efficacy in reducing joint damage. This approach presents a targeted and inflammation-responsive strategy for OA therapy, capitalizing on the dynamic immunomodulatory capabilities of macrophages to foster joint health and functionality.

Similarly, immunomodulatory NPs conjugated with peptide antigens relevant to joint pathology have the potential to modulate inflammatory flares associated with autoimmune joint disorders, such as rheumatoid arthritis. These NPs, termed calcitriol-loaded NPs, encapsulate calcitriol, an immunoregulatory agent, within a poly(lactic-co-glycolic acid)-poly(ethylene glycol)-maleimide matrix [92]. Through targeted delivery of both calcitriol and joint-relevant peptide antigens, these calcitriol-loaded NPs were found to induce phenotypic changes in dendritic cells, reduce the production of pro-inflammatory cytokines, and enhance the expression of immune regulatory genes, such as cytotoxic T-lymphocyte associated protein 4. In animal models of autoimmune joint disorders, calcitriol-loaded NPs effectively reduced disease severity, while preserving joint integrity and modulating immune responses without eliciting generalized immune suppression. This study demonstrated the potential of immunomodulatory NPs to selectively target inflammatory processes and alleviate autoimmune joint disorders, thereby providing a flare-responsive strategy for personalized therapy.

Furthermore, the modulation of the bone microenvironment mediated by NPs was investigated as a strategy to ameliorate OA. Fibronectin (FN)-coated NPs responsive to reactive oxygen species (ROS) were designed to scavenge ROS, inhibit the production of inflammatory cytokines, and promote osteogenesis for effective OA treatment [94]. The developed NPs loaded with azabisdimethylphoaphonate-terminated phosphorus dendrimers (G4-TBP) exhibited stability under physiological conditions and were internalized by macrophages through FN-mediated targeting. In animal models of OA, these NPs significantly reduced synovitis, inhibited cartilage matrix degradation, and promoted osteogenic differentiation for bone repair. NP-mediated modulation of the bone microenvironment offers a comprehensive approach to OA therapy by integrating anti-inflammatory and antioxidative properties, effectively addressing both the inflammatory and degenerative aspects of the disease.

Responsive nanosystems designed for cartilage regeneration and immunomodulation hold considerable promise for the treatment of OA and related joint disorders. From macrophage membrane-coated NPs to immunomodulatory peptide hydrogels and bone-targeted NPs, these cutting-edge strategies exploit the dynamic characteristics of the joint microenvironment to facilitate targeted and multifunctional therapies [95]. Responsive nanosystems provide a personalized and comprehensive strategy for enhancing cartilage regeneration and improving the quality of life for OA patients by concurrently modulating inflammatory responses, promoting tissue regeneration, and strengthening joint integrity.

### Nanosystem-based immunomodulation for soft tissue regeneration

Soft tissue regeneration, particularly in the context of diabetic wound management, faces substantial challenges due to compromised healing mechanisms and the complicated microenvironment associated with diabetes. The impaired healing processes inherent to diabetic wounds pose formidable obstacles to achieving successful tissue regeneration [96–98]. In light of these challenges, researchers have explored innovative strategies that harness the potential of responsive nanosystems to fulfill the dual goals of immunomodulation and tissue repair.

These approaches utilize the capabilities of responsive nanotechnology to provide a promising solution to the complex pathophysiology of diabetic wounds. Through precise modulation of immune response and targeted delivery of therapeutic agents, nanosystems present significant potential for promoting tissue regeneration in environments that would otherwise impede healing. This interdisciplinary approach not only addresses the underlying immunological dysregulation but also facilitates tissue repair, offering renewed hope for improved clinical outcomes in the management of diabetic wounds.

One notable approach entails the development of biomimetic hydrogels decorated with nanozymes, such as MnCoO-based polydopamine/conducting polymer hydrogels (MnCoO@PDA/CPH) [99]. These hydrogels exhibited H_2_O_2_-activated oxygenation ability and were designed to mimic the mechanical and electrical properties of skin. In preclinical studies using diabetic wound models, these hydrogels have demonstrated remarkable efficacy in wound healing by promoting re-epithelialization, collagen deposition, and angiogenesis, underscoring their potential as therapeutic wound dressings for managing diabetic wounds, where conventional methods frequently fall short due to compromised immune response and a challenging wound microenvironment.

Another promising strategy for diabetic wound repair involves the utilization of MXene-M2-exosome nanocomposites, exemplified by MXene-M2 macrophage exosome (Exo) nanohybrids (FM-Exo) [100]. These nanocomposites effectively counteract immune suppression induced by high glucose levels through the induction of optimized M2 macrophage polarization, leading to enhanced proliferation and migration of fibroblasts, as well as the angiogenic capacity of endothelial cells, crucial components in the wound healing process. In diabetic wound models, FM-Exo has been demonstrated to proficiently regulate macrophage polarization, suppress inflammation, promote angiogenesis via VEGF secretion, and ultimately expedite the healing process. The ability of these nanocomposites to modulate the immune response and promote tissue repair emphasizes the potential for innovative therapeutic strategies in managing diabetic wounds.

In addition to hydrogel-based and nanocomposite approaches, composite structures like Mn-Si-chitooligosaccharide-based mesoporous silica NPs (COS@Mn-MSN) present unique opportunities for regulating the wound microenvironment and inhibiting skin fibrosis. COS@Mn-MSN has been shown to effectively reduce inflammation and promote proper collagen deposition via scavenging intracellular ROS and shifting macrophage polarization [101]. Furthermore, these composites downregulated TGF-β1-mediated signaling pathways, thereby inhibiting the formation of excessive skin fibrosis. The multifaceted mechanism of action exhibited by COS@Mn-MSN holds promise for scarless wound therapy, addressing a critical aspect of soft tissue regeneration. Collectively, these innovative nanosystems signify substantial advancements in the field of wound management, offering hope to improve diabetic wound healing and soft tissue regeneration [102–104].

### Correlation between nanomedicine and neurotransmitters

The interaction between the nervous system and fibrous connective tissue plays a vital role in tissue repair, with neurotransmitters acting as key regulatory agents in this intricate process [105, 106]. Neurotransmitters such as acetylcholine, dopamine, and serotonin, while primarily linked to neuronal communication, also exert significant influence on the cellular and molecular mechanisms that underpin tissue repair and regeneration [107, 108]. The development of nanomedicine offers advanced strategies to precisely modulate neurotransmitter activity, thereby enhancing therapeutic outcomes in tissue repair. NPs can be engineered to deliver neurotransmitter modulators or analogs directly to injury sites, enabling precise control over the repair process. This targeted delivery aligns neurotransmitter activity with the body’s natural healing processes, reducing the risk of systemic side effects. For example, NPs can be designed to release neurotransmitters in response to specific environmental stimuli at the injury site, such as changes in pH or temperature, providing a localized and responsive therapeutic approach [109]. This strategy not only optimizes the timing and site-specific release of neurotransmitters but also cultivates a microenvironment conducive to tissue repair by promoting cellular activities, including proliferation, differentiation, and ECM production.

Additionally, the capabilities of nanomedicine extend to the modulation of neurotransmitter receptors on cells within fibrous connective tissue. Nanostructured scaffolds that incorporate neurotransmitter-releasing systems can be employed to establish a regenerative microenvironment that replicates the physiological conditions essential for effective tissue repair [110]. These scaffolds can be engineered to release neurotransmitters in a controlled and sustained manner, thereby supporting long-term tissue regeneration. Furthermore, nanomedicine has the potential to influence neurotransmitter synthesis, release, and degradation, thereby indirectly modulating the overall repair process. For instance, NPs delivering growth factors or cytokines can alter the expression of enzymes involved in neurotransmitter metabolism, thus enhancing nerve regeneration, and facilitating the integration of fibrous connective tissue with newly formed neural networks [111, 112]. Beyond therapeutic applications, nanomedicine also provides sophisticated tools for investigating neurotransmitter dynamics during tissue repair. NP-based imaging agents enable real-time monitoring of neurotransmitter release and activity in vivo, offering valuable insights into the interaction between the nervous system and fibrous connective tissue throughout the healing process [113]. By leveraging these capabilities, nanomedicine not only deepens our understanding of tissue repair mechanisms but also paves new pathways for developing innovative therapies that exploit the regulatory power of neurotransmitters. The integration of nanomedicine with neurotransmitter-based approaches thus holds significant potential for advancing tissue repair and regeneration, while promising improved clinical outcomes across various medical conditions.

### Nanosystem-based immunomodulation for nerve repair and regeneration

Nerve repair and regeneration are complex processes, particularly in the context of injuries such as spinal cord injury (SCI) and ischemic stroke. In these conditions, the immune response profoundly shapes the microenvironment and determines the trajectory of tissue repair [114, 115]. Nanosystems possess the potential to shift the balance toward regeneration by functionally targeting immune cells and modulating signaling pathways, offering new hope for patients with nerve injuries. This innovative strategy represents a significant advancement in efforts to harness the body’s intrinsic healing mechanisms for neural function restoration, promising improved outcome and quality of life for individuals affected by debilitating conditions like SCI and ischemic stroke.

A variety of nanomaterials, including magnesium/aluminum layered double hydroxide (Mg/Al-LDH) NPs, DNase-loaded NPs, carbon nanotube scaffolds, sulfated polysaccharide-based nanocarriers, and polyphenol-metformin NPs, have been developed to target different aspects of the immune response and facilitate nerve repair [116]. One approach to promote nerve repair involves the use of Mg/Al-LDH NPs, which have demonstrated the ability to accelerate neural stem cell migration, differentiation, and action potential generation [116]. These NPs target TGF-β receptor 2, a key regulator of inflammation and neural regeneration. By inhibiting inflammatory responses while promoting the proliferation of neural precursor cells, Mg/Al-LDH NPs foster a supportive microenvironment for nerve repair. Additionally, these NPs modulate the activity of microglia and bone marrow-derived macrophages by shifting their phenotype from pro-inflammatory (M1) to anti-inflammatory (M2). Through targeted delivery of neurotrophic factors such as neurotrophin-3, Mg/Al-LDH NPs enhance the functional recovery in injured nerves. This multifaceted approach highlights the potential of Mg/Al-LDH NPs to promote both structural and functional regeneration of neurons following injury.

An alternative strategy entails the utilization of DNase-loaded NPs to target the cyclic guanosine monophosphate–adenosine monophosphate synthase (cGAS)-stimulator of interferon genes (STING) pathway, which serves as a crucial mediator of neuroinflammation following ischemic stroke [117]. These NPs combine the enzymatic activity of DNase with the inhibitory properties of STING antagonists to reduce the accumulation of immunogenic DNA fragments and alleviate the chronic inflammatory cascade. DNase-loaded NPs provide a synergistic approach to ameliorating neuroinflammation and promoting tissue repair by modulating the cGAS-STING axis. Moreover, employing nanocarriers allows for targeted delivery of therapeutic agents to the site of injury, thereby minimizing off-target effects, while enhancing efficacy. Through a combination of biochemical and biophysical signals, DNase-loaded NPs hold promise for improving the prognosis of ischemic stroke by dampening neuroinflammation and facilitating tissue repair.

Carbon nanotube scaffolds represent another innovative approach for modulating immune responses in the context of nerve repair [118]. By interfacing with human dendritic cells, these scaffolds influence the immunogenic profile of activated immune cells, potentially reducing inflammation, and promoting neural regeneration. Through a combination of phenotype analysis, microscopy, and transcriptional profiling, researchers have elucidated the immunomodulatory effects of carbon nanotube scaffolds. These findings emphasize the importance of comprehending the interactions between nanomaterials and immune cells for nerve repair [119, 120]. Researchers aspire to develop scaffolds that effectively guide tissue growth and organization while minimizing adverse immune responses, leveraging the immunomodulatory properties inherent to carbon nanotubes.

### Clinical trials and preliminary effects of promising nanomedicine

Recent developments in nanomedicine have yielded several promising candidates currently undergoing clinical trials, particularly in the field of tissue repair and regeneration. They exploit the unique properties of NPs, such as precise therapeutic delivery, modulation of the microenvironment, and interactions with cellular pathways, to enhance tissue healing processes.

A prominent example is nano-hydroxyapatite (nHA), which is currently being investigated for its potential applications in bone regeneration. This nanomaterial, closely resembling the mineral composition of bone, has exhibited significant efficacy in promoting osteogenesis, increasing bone density, and integrating with existing bone tissue [60, 83]. Preliminary clinical trials have indicated that nHA can enhance bone healing in patients with fractures or bone defects, underscoring its strong potential for clinical application in orthopedic treatments [121].

Another noteworthy advancement in nanomedicine is the liposomal formulation of growth factors, specifically designed to accelerate wound healing. These liposomal nanocarriers are engineered to protect and deliver growth factors directly to the wound site, thereby enhancing angiogenesis and collagen deposition [122, 123]. Early-phase clinical trials have reported that these formulations significantly shorten healing times in chronic wounds, such as diabetic ulcers, suggesting their substantial clinical potential in wound care management [123].

NP-based gene therapy is also making significant progress in clinical trials, particularly concerning nerve repair [124]. These NPs are designed to deliver genes encoding neurotrophic factors, which facilitate nerve regeneration and repair. Preliminary results from clinical trials suggest that this approach can enhance nerve regeneration in patients with peripheral nerve injuries, leading to improved sensory and motor function recovery [125, 126]. This emphasizes the potential of NP-mediated gene therapy for addressing nerve damage.

## Conclusions and perspective

Tissue defects pose substantial challenges to healthcare systems worldwide, necessitating the development of innovative approaches for effective regeneration. The immune microenvironment within these defects plays a pivotal role in orchestrating tissue repair processes, and dysregulation of immune responses can impede optimal healing. Microenvironment-responsive nanomedicine has emerged as a promising strategy for the precise modulation of the immune microenvironment, facilitating targeted delivery of immunomodulatory agents to promote tissue regeneration.

In this review, we elucidate the distinctive features of the immune microenvironment within tissue defects and investigate the potential of nanomedicine for immunomodulation in tissue regeneration. We elaborate on the design principles and functional properties of immunomodulatory nanosystems, highlighting their ability to respond to environmental cues and modulate immune cell behavior effectively. Moreover, we assess the challenges and opportunities associated with the clinical translation of nanomedicine, emphasizing the necessity for interdisciplinary collaboration and personalized approaches to optimize therapeutic outcomes.

Microenvironment-responsive nanomedicine holds great promise for advancing tissue regeneration by harnessing the complex interactions among immune cells, growth factors, and ECMs. Through precisely targeting the immune microenvironment, nanomedicine provides a powerful platform for enhancing tissue repair processes while minimizing off-target effects and systemic toxicity [127–129]. Applying these principles to tissue regeneration necessitates the modulation of immune responses to foster a regenerative-friendly microenvironment. Responsive nanomedicine can accomplish this via the targeted delivery of immunomodulatory agents directly to sites of tissue damage. For example, NPs loaded with anti-inflammatory drugs can suppress excessive inflammation, while those carrying growth factors can stimulate tissue repair [73, 130]. Responsive nanomedicine ensures optimal regulation of the immune response throughout the entire regeneration process via precisely controlling the release kinetics and dosage of these agents.

The design of responsive nanosystems for tissue regeneration involves a deep understanding of both the physicochemical properties of NPs and their therapeutic payloads. The properties of NPs, including size, shape, surface chemistry, and responsiveness to environmental stimuli, govern their interactions with the immune system and their capacity to target specific tissues [131]. Stimuli-responsive NPs, for example, can release therapeutic agents in response to various cues such as pH, temperature, and enzymatic activity, thereby enabling spatiotemporal control over drug delivery [132]. Furthermore, the choice of therapeutic payloads, ranging from small molecules to nucleic acids, proteins, and cells, is pivotal in the modulation of immune responses that promote tissue regeneration [133]. Responsive nanosystems can be functionalized to address the unique challenges of tissue regeneration across various organs and disease contexts by effectively integrating these design considerations.

In the realm of tissue regeneration, NPs loaded with growth factors exhibit promising potential in facilitating the regeneration of both bone and cartilage tissues. Similarly, in neurology, these NPs serve as carriers for neurotrophic factors, stimulating nerve growth and facilitating repair processes. Furthermore, the application of responsive nanosystems extends to the development of artificial scaffolds that mimic the ECM, thereby guiding tissue growth in regenerative medicine frameworks. Moreover, the targeting capability of NPs toward specific cell types, such as stem cells and immune cells, augments their therapeutic efficacy in tissue regeneration endeavors. These advancements, documented in various studies [87, 88, 134, 135], underscore the versatility and adaptability of microenvironment-responsive nanomedicine in developing interventions tailored to meet the distinctive requirements of diverse tissue types and pathological conditions, thus providing novel strategies for regenerative medicine, and improving patient outcomes.

Shortly, a more profound comprehension of the molecular mechanisms underlying nanomedicine-mediated immunotherapy, coupled with advancements in nanotechnology, will inspire researchers to develop more effective immune-targeting strategies. Central to this progression is the precise manipulation of NP interactions within diverse cellular environments. Advances in materials design emphasize the development of multifunctional NPs capable of delivering therapeutic payloads and dynamically responding to local tissue signals [53, 136, 137]. This adaptability is crucial for designing interventions that meet the evolving needs of damaged tissues and facilitate effective regeneration. Additionally, incorporating bioactive molecules and smart polymers into NP frameworks enhances biocompatibility and ensures sustained therapeutic efficacy amidst intricate cellular responses [138].

Furthermore, investigations into cellular microenvironments will increasingly rely on advanced imaging techniques and computational models to elucidate the interactions between NPs and cells. This enhanced understanding will clarify how NPs modulate immune responses, mitigate inflammation, and promote tissue-specific regeneration pathways. Moreover, NPs with tunable surface properties, such as charge and hydrophobicity, enable precise targeting of specific cell populations or subcellular compartments, thereby optimizing therapeutic outcomes while minimizing off-target effects [139, 140]. Exploiting these capabilities will be crucial in overcoming current limitations in tissue regeneration, particularly in compromised or dysregulated microenvironments.

In addition to advancements in materials innovation, future research in tissue regeneration will explore bioinspired strategies that mimic natural ECMs to direct cellular behavior and tissue organization. NPs engineered to replicate ECM components, such as collagen or elastin-like peptides, provide structural support and biochemical signals that enhance cell adhesion, migration, and differentiation [57]. This biomimetic approach promotes the integration of transplanted cells into host tissues and facilitates the development of functional tissue substitutes that closely resemble their native counterparts in both structure and function. Additionally, incorporating growth factors or nucleic acid-based therapeutics within ECM-mimetic NPs further augments regenerative capacity by stimulating endogenous repair mechanisms and orchestrating tissue remodeling processes [10].

The future of tissue regeneration and NP design is marked by the convergence of interdisciplinary approaches, technological innovations, and a dedicated effort to comprehend and leverage the complexities of the cellular microenvironment. By capitalizing on these synergies, researchers can propel transformative therapies that restore tissue functionality, enhance patient outcomes, and improve overall quality of life. Nevertheless, several unresolved challenges should be addressed to effectively translate responsive nanomedicine for tissue defects into clinical practice. (1) Achieving precise control over the spatiotemporal release of therapeutic agents in complex biological environments remains a formidable challenge. To tackle this issue, advanced stimuli-responsive nanocarriers should be developed that can respond to multiple biological signals such as pH, temperature, and enzyme activity. Additionally, computational modeling and machine learning may be employed to predict and optimize release kinetics tailored for specific tissues and conditions, thereby enhancing the effectiveness and precision of these nanomedicine interventions. (2) Ensuring the safety and biocompatibility of nanomaterials, along with their degradation products, is essential for clinical translation. Comprehensive in vitro and in vivo studies are necessary to evaluate the toxicity profiles, immunogenic responses, and long-term biocompatibility of these materials. Furthermore, developing biodegradable nanomaterials that decompose into non-toxic byproducts while being efficiently cleared from the body will further enhance their safety profile, facilitating effective clinical implementation. (3) Efficiently targeting specific cell types such as stem cells and immune cells without adversely affecting non-target cells presents significant challenges. Surface modification techniques can be employed to functionalize NPs with ligands or antibodies specifically designed to bind receptors on target cells. Moreover, utilizing dual-targeting approaches could enhance specificity and reduce off-target effects, thereby improving both precision and efficacy in nanomedicine treatments. (4) Biological barriers including ECMs and cellular membranes may hinder delivery efficiency alongside therapeutic effectiveness in nanomedicine applications. To overcome these obstacles, NPs should be engineered with characteristics and incorporate cell-penetrating peptides or other translocation-enhancing features. Additionally, exploring novel carrier systems capable of modulating ECM will facilitate deeper tissue penetration, ultimately improving delivery for effective treatment outcomes using nanomedicine. (5) Ultimately recognizing that tissue defects arise from various pathological factors underscores the necessity for multifunctional immunomodulatory nanomedicine. Furthermore, reducing synthesis costs will significantly promote successful clinical applications aimed at treating various forms of tissue defects.

## Data Availability

The datasets used or analyzed during the current study are available from the corresponding author on reasonable request.

## Abbreviations

cGAS: Cyclic guanosine monophosphate-adenosine monophosphate synthase
ECM: Extracellular matrix
EPR: Enhanced permeability and retention
Exo: Exosome
FM-Exo: MXene-M2 macrophage exosome nanohybrids
FN: Fibronectin
GHANPs: Glycosylated nano-hydroxyapatites
G4-TBP: Azabisdimethylphoaphonate-terminated phosphorus dendrimers
IL-6: Interleukin-6
MnCoO@PDA/CPH: MnCoO-based polydopamine/conducting polymer hydrogels
Mg/Al-LDH: Magnesium/aluminum layered double hydroxide
nHA: Nano-hydroxyapatite
NPs: Nanoparticles
OA: Osteoarthritis
ROS: Reactive oxygen species
SCI: Spinal cord injury
STING: Stimulator of interferon genes
TNF-α: Tumor necrosis factor-α
TGF-β: Transforming growth factor-β
VEGF: Vascular endothelial growth factor
